# NIR-II Fluorescence Imaging of Skin Avulsion and Necrosis

**DOI:** 10.3389/fchem.2019.00696

**Published:** 2019-10-22

**Authors:** Yizhou Li, Xiang Hu, Wanrong Yi, Daifeng Li, Yaqi Guo, Baiwen Qi, Aixi Yu

**Affiliations:** Department of Orthopedics Trauma and Microsurgery, Zhongnan Hospital of Wuhan University, Wuhan, China

**Keywords:** NIR-II fluorescence imaging, skin avulsion, necrosis, apoptotic cells, caspase-3

## Abstract

Skin avulsion is commonly seen in individuals exposed to heavy shearing forces. Subcutaneous tissue detachment and bone fractures usually accompany skin avulsion. Thus, the estimation of the extent of damaged tissue is very important. Currently, the viability of skin and subcutaneous tissue is determined by clinical observations, and these observations always underestimate the true extent of the avulsed skin. Herein, we synthesized an innovative probe, CH1055-GRRRDEVDK (CH1055-GK), which can specifically bind to caspase-3 so as to image skin avulsion and define necrotic regions. Our uptake and binding affinity tests in apoptotic cells and evaluation of the probe *ex vivo* and *in vivo* showed that the probe has a strong ability to bind caspase-3 in skin avulsion models and that it vividly detected the necrotic area in avulsed skins. Furthermore, the increased fluorescence intensity of the probe in the avulsed skin showed a larger affected area than that determined by clinical observations in live mice. Consequently, our results indicated that observation of the caspase-3-targeted probe CH1055-GK via NIR-II imaging allowed the clear detection of skin avulsion in subjects, indicating its potential as an imaging tool for the early diagnosis of skin avulsion and the determination of necrotic margins.

## Introduction

The incidence of skin avulsion has increased dramatically due to traffic accidents and machine accidents in modern industry (Tosti and Eberlin, 2018; Weinand, 2018). The large area of skin avulsion injuries, combined with severe shock or fractures, causes severe soft tissue crushing and massive blood loss (Latifi et al., 2014; Weinand, 2018). The skin and subcutaneous tissues were detached from the underlying fasciae and musculoskeletal structures. The clinical treatment of these injuries is time-consuming and is often delayed so that the avulsed flaps easily develop into ischemia and ultimately into secondary skin necrosis because of the damage of blood circulation. Avulsion injuries may lead to high morbidity and even mortality (Olingy et al., 2017; Sakai et al., 2017). It is essential to identify the viability of avulsed skin as accurately as possible in order to efficiently improve the survival rate of avulsed skins. Clinicians usually determine the viability of avulsed skins through examination of cutaneous bleeding, skin color, pressure reactions, and microcapillary refills. However, these clinical symptoms are often not accurate enough to predict the degree of skin avulsion injuries (Gagnier et al., 2013; Latifi et al., 2014; Sen et al., 2017), and this may result in extensive excision of avulsed skins and soft tissue or secondary infection and necrosis. Therefore, imaging techniques have become necessary to assist in the evaluation of the viability of avulsed skin and necrosis. Although current clinical imaging techniques, including computed tomography (CT) and magnetic resonance imaging (MRI), have been used to assess, the location, extent, and severity of acute tissue injuries or applied to detect skin inflammation and skin cancer (MacFarlane et al., 2017; Ueno et al., 2018), they have rarely been involved in evaluating skin avulsion and necrosis. As such, it is meaningful to explore promising imaging techniques that can assist in the early assessment of skin avulsion and necrosis.

In recent years, the significant benefits of molecular imaging have been demonstrated in targeted surgery with preoperative molecular diagnostic screening and fluorescence image-guided surgery (Reinsmith et al., 2013; Iglesias et al., 2014; Walton et al., 2017; Li et al., 2019; Zeng et al., 2019). NIR fluorescence imaging can provide physiological and pathological information at the cellular and tissue levels that can be applied in biology and medicine to diagnose or treat clinical diseases (Ntziachristos et al., 2005; Pansare et al., 2012; Hong et al., 2015; Lin et al., 2019). Based on fluorescence emission, the NIR imaging window is divided into two different imaging windows (the first, NIR-I, 750–900 nm; the second, NIR-II 1,000–1,700 nm) (Hong et al., 2014; Antaris et al., 2017). In comparison with the NIR-I window, the NIR-II window has dramatically improved imaging quality and signal-to-noise ratio due to minimal light scattering and autofluorescence. NIR-II imaging has great potential for deep-tissue imaging with improved spatial resolution and increased contrast (Smith et al., 2009; Sun et al., 2016). At present, a series of fluorescent NIR-II probes including small molecules and quantum dots is being actively employed for cerebral, vascular, and lymphatic imaging and for tumor imaging-guided surgery (Hong et al., 2012; Antaris et al., 2016; Sun et al., 2017b, 2018; Yang et al., 2018; Zeng et al., 2018). In summary, the applications of NIR-II molecular probes are having a direct impact on the field of biomedicine and clinic research.

At the beginning of skin avulsion, the soft tissue is severely crushed, and massive blood vessels are suddenly destroyed so that it presents a level of hypoxic and ischemic damage, and thus, cell metabolism is converted to an anaerobic condition (Siemionow and Arslan, 2004). Moreover, following hypoxic and ischemic damage, there is a major phase of cell death involved in cell inflammation and apoptosis, which is regulated by caspase-3 and activated in intrinsic pathways (Taylor et al., 2008; Rogers et al., 2017; Strzyz, 2017; Ponder and Boise, 2019). Caspase-3 is not only a primary pivot or trigger in regulating the initiation and propagation of the early stage of apoptotic concatenation but has a specific recognition site, DEVD (Wu et al., 2019). Notably, this tetrapeptide motif can be bound specifically to the active caspase-3, and many DEVD peptides have been reported to be used to evaluate caspase-3 activation (Srinivasula et al., 2001; Casares et al., 2005; Scabini et al., 2011; Sun et al., 2017a).

Herein, we firstly used a caspase-3-targeting NIR-II probe (CH1055-GK) to detect the skin avulsion and necrosis of avulsed skin in the early trauma stage. According to our results, CH1055-GK was investigated for the accurate detection of skin avulsion and necrosis in skin avulsion mouse models. These positive results for CH1055-GK also make it a promising caspase-3-targeted NIR-II probe for clinical translation.

## Materials and Methods

### Synthesis and Characterization of NIR-II Probes

All chemical reagents were purchased from commercial sources (such as Aldrich, Ameresco, Energy Chemical) and used without further purification unless otherwise noted. HPLC was performed on a Dionex HPLC System (Dionex Corporation) equipped with a GP50 gradient pump and an in-line diode array UV-Vis detector. The peptide GK (1.112 DKa) was purchased from GL Biochem (Shanghai) Ltd. The previous literature can be referred to for details of the synthesis of CH1055 (0.969 KDa) (Antaris et al., 2016). The synthesis of CH1055-GK or CH1055-PEG (as a control) was carried out as follows. A solution of CH1055 (100 μg, 0.103 μmol) in dry DMF (*N, N*-Dimethylformamide) was stirred for 10 min, and then NHS (N-hydroxysuccinimide) (59.27 μg, 0.515 μmol) and EDCI (1-ethyl-3-(3-dimethylaminopropyl)carbodiimide) (98.73 μg, 0.515 μmol) were added to the mixture and stirred for 2 h under N_2_ protection. Peptide GK (581.44 μg, 0.515 μmol) or COtBu-PEG_4_-NH2 (Polyethylene glycol, 0.989 KDa, Ponsure Biological) (165.52 μg, 0.515 μmol) and DIPEA (*N, N*-diisopropylethylamine) (15 μL) was then added to the reaction solutions and stirred overnight. The final product was purified by HPLC. HPLC condition: eluent: water/acetonitrile (containing 0.1% TFA, from 70 to 95%); over 22 min with a flow rate of 1 mL min^−1^ and detection at 254 nm.

### Cell Culture and Cell Apoptosis Induction

Human HACAT, HUVEC, and HSF cells (iCell Bioscience Inc.) were cultured in DMEM (Dulbecco's Modified Eagle Medium)/DMEM-F12 (Dulbecco's Modified Eagle Medium/Nutrient Mixture F-12, Hyclone) supplemented with 10 or 15% FBS (Fetal Bovine Serum, VWR, Radnor, PA) and antibiotics (100 U mL^−1^ penicillin) at 37°C incubator with 5% CO_2_. The cells were fed every 1–2 days and sub-cultured when they reached 70–80% confluence. For apoptosis induction, cells were cultured in 6-wells for 24 h, and then 2 mL of culture medium mixed with 20 μmol/L Adriamycin (Thermo Fisher Scientific Inc.) was added to each of the six wells and cultured for 6 and 12 h at 37°C incubator with 5% CO_2_ (Crowley et al., 2016).

### Flow Cytometric Detection of Apoptosis

After treatment with Adriamycin, the culture medium was removed, and then cells were washed twice with pre-cooling PBS (Phosphate-buffered Saline, Gino Biomedical Technology). Next, cells were harvested and resuspended in 300 μl binding buffer containing 5 μl Annexin V-FITC (Fluorescein Isothiocyanate, Sungene Biotech) as well as 5 μl PI (Propidium Iodide, Sungene Biotech), and were then incubated at room temperature (RT) in the dark for 15 min. Finally, we ran each cell sample, and the data were quantified by flow cytometer.

### Cell Uptakes and Binding Affinity Studies

The CH1055-GK probe was dissolved in PBS with 5% dimethyl sulfoxide (DMSO, Gino Biomedical Technology) to a concentration of 0.5 nm μL^−1^. For the uptake study, apoptotic cells were randomly assigned to each group and incubated with CH1055-GK (2 nM probe per 200 μL PBS per sample) in 6-well cell culture plates (Costar, Corning, NY). For the blocking group, 1 h incubation with peptide GK (200 nM) was performed before the incubation with CH1055-GK. At different incubation times (0–24 h), cell samples were washed three times with cold PBS, and then the apoptotic cells were collected using an enzyme-based cell detachment solution in the PCR tubes (250 μl). Furthermore, the PCR tubes were centrifuged for 3 min at 400 r/min. The supernatant was then removed using a NIR-II system purchased from Suzhou NIR-Optics Technologies (Suzhou, China). The binding affinity was tested by preparing different concentrations of peptide GK in PBS (1 × 10^−3^-10^−9^ M per 200 μL PBS per sample) as the blocking agents for CH1055-GK. Apoptotic cells were incubated with the blocking agent for 1 h and then incubated with CH1055-GK (2 nM probe per 200 μL PBS per sample) for 8 h. The subsequent steps were performed as described above for the cell-uptake experiment. Finally, NIR images were collected with a NIR-II system.

### Introduction of the Skin Avulsion Model

Female BALB/c (nu/nu) mice were obtained from Charles River Laboratories (Beijing Vital River Laboratory Animal Technology Co., Ltd.) and kept under sterile conditions before establishing the skin avulsion model. The animals were housed in cages with sawdust bedding in an air-conditioned room and were fed a standard laboratory diet.

Twenty female BALB/c (nu/nu) mice (aged 7–8 weeks, 20–25 g) were used to establish the skin avulsion mouse models according to previous protocols (Milcheski et al., 2013; Chin et al., 2017). Anesthetized mice were fixed in a stable plane. After removing the hair on the dorsal side of the mouse with an electric razor, the surgical region was disinfected by 75% ethyl alcohol. A full-thickness (4 × 5 cm) skin wound was created on the dorsal skin. Afterward, all of the skin was separated from the subcutaneous tissue with a scalpel handle, and then the skin was affixed with a small wooden rod with one hole. The skin was then carefully pulled with a tension machine (15 N) for 10 s. Finally, the full-thickness injured skin was intermittently sutured back to the primordial area. The same method was used for all mice. All mice received an injection of the same amount of saline before they were placed back in the cages, and the respiration and body temperature of the mice were recorded. The mice were housed in separate cages and prepared for NIR-II imaging experiments.

### *In vivo* and *ex vivo* NIR-II Imaging of Skin Avulsion

In order to evaluate the NIR-II imaging of skin avulsion *in vivo* study, 100 μ*L* CH1055-GK (0.5 nM μ*L*^−1^) was administered into the mice via tail-vein injection as the experiment group. 100 μ*L* CH1055-GK blocking (with 1 μ*M* blocking agent) and 100 μ*L* CH1055-PEG were also injected into the mice as the blocking or control group respectively. All of mice were skin avulsion model and were randomly divided into three groups (experimental group, control group and blocking group) (*n* = 3 for each group). During injection and imaging, mice were anesthetized using a 2 L min^−1^ oxygen flow with 2% isoflurane. In *ex vivo* imaging, fresh avulsed skin samples (~3 mg wet weight) were obtained with surgical scissors, and then these samples, randomly allotted to the experimental, blocking, and control groups, were incubated with CH1055-GK, blocking agent, or CH1055-PEG, respectively. The incubation protocol was the same as that used in the cell uptake experiment. For imaging in the NIR-II window, a 1,000 LP filter was used for obtaining fluorescence imaging, and the suitable exposure time was 20 ms. Mice and samples were imaged at different time points (2, 6, 12, 24 h).

### MRI of Mice

All subjects were scanned on a Bruker 7T/20 cm MRI system (Ettlingen, German), using a body coil with a diameter of 72 mm to transmit a radio-frequency pulse and a quadrature surface coil with a diameter of 40 mm to receive signals. During the MRI scan, mice were anesthetized with 1.5–2.5% isoflurane. MRI scout films were used to confirm that the knees were parallel to the axis of the femur and that the acquisition of slices was directly perpendicular to the articular cartilage surfaces of the medial tibial plateau. All images were acquired using a conventional T2^*^-weighted gradient-recalled echo (GRE) sequence with a flip angle of 180 degrees, a repetition time of 500 ms, and an echo time of 12 ms. The scan time was 16 min, using a 3.6 cm field of view and a slice thickness of 1 mm. After the MRI scan, the data were processed by MRI software version 1.40.

### *In vitro* and *in vivo* Cytotoxicity of CH1055-GK

The cytotoxicity of CH1055-GK was evaluated by cell counting kit-8 (CCK-8) assay (Thermo Fisher Scientific Inc.) in human HUVEC, HSF, and HACAT.

Firstly, 100 μL of DMEM/F12 supplemented with 10% FBS was added to each 96-well, and then these cells were cultured overnight at 37°C incubator with 5% CO_2_. Secondly, cells were incubated with CH1055-GK at different concentrations (0, 2, 4, 8, 16 μmol/L) from 2 to 24 h. Thirdly, 10 μL of CCK-8 solution was added to each 96-well and incubated for another 2 h. After adding 200 μL of DMSO (VWR, Radnor, PA) and shaking for 15 min at low-speed (50 rpm), cell viability was determined using an auto ELISA detector (DR-200Bs, Diatek) at 450 nm. All samples were repeated five times. For *in vivo* cytotoxicity, the probe CH1055-GK (10 mg kg^−1^) was injected into mice through the tail vein as the experimental group, and an equal volume of PBS was administrated to the control group. After that, mice were euthanized, and their major organs (Heart, Liver, Spleen, Stomach, Lung, Kidney, Brain, and Intestine) were harvested 24 h post-injection, and H&E staining was performed to evaluate toxicity *in vivo*.

### Immunofluorescence Staining

For the immunofluorescence staining, the cell suspension was added to a glass coverslip and incubated at 37°C incubator with 5% CO_2_ for 2 h, followed by washing in PBS for 3 × 5 min. Cells were then placed into 4% paraformaldehyde for 30 min at RT and washed 3 times with PBS. Next, cells were incubated with 50–100 μl permeabilization buffer (0.5% Triton X-100 in PBS) for 10 min at RT and washed 3 times with PBS for 5 min each. Cells were then incubated with 3% hydrogen peroxide solution for 20 min in the dark at RT, followed by three PBS washes. After that, 5% BSA (Bovine Serum Albumin, Roche) was used to block cells for 1 h at room temperature. Primary mouse anti-human antibody (1:1,000 dilution, Abcam, ab49822) was added to each well and incubated overnight at 4°C. After rinsing with PBS, cells were incubated with CY3-conjugated goat anti-mouse antibody (1:50 dilution, Aspen, AS-1109). After another incubation for 2 h at 37°C, the cells were again washed three times with PBS and treated for 5 min with 50–100 μl DAPI (No. AS1075; Aspen Biotechnology) to stain the cell nuclei. Finally, the results were examined under a fluorescence microscope (MircoPublisher, Q-IMAGING).

### Statistical Analysis

The NIR fluorescence signals were quantitated with ImageJ 1.38x software (National Institutes of Health, Bethesda, MD). The region-of-interest (ROI) was manually defined around the joint, and the average signal within the ROI was obtained. The data are given as the mean ± SD (standard deviation). Statistical analysis was performed using a two-tailed Student's *t*-test. Statistical significance was assigned for *P*-values < 0.001, 0.01, and 0.05.

## Results and Discussion

### Design and Detection Mechanism of CH1055-GK

As previously reported, skin avulsion leads to damage to soft tissues and blood vessels, which may be associated with different biological processes including ischemia and hypoxia (Ballestin et al., 2018). Therefore, apoptosis pathways mediated by caspase-3 are induced and may exert an immediate influence on the viability of the avulsed skin (Liu et al., 2015). In recent years, probes have been used to target and monitor the activity of caspase-3 (Shaulov-Rotem et al., 2016), but no studies have demonstrated a small-molecule probe that can be used to discover skin avulsion and even to distinguish necrotic regions via targeting caspase-3. Hence, we hypothesized that if a small molecular probe is good enough to enter the skin to detect skin avulsion and necrosis. On the one hand, applications of the small-molecule NIR-II fluorophore CH1055 have reportedly made great progress in molecular tumor imaging and image-guided surgery (Zhang et al., 2016; Yang et al., 2017). Moreover, several unique advantages of CH1055, including its low auto-fluorescence, high signal–background ratio, and deeper tissue penetration, may make it suitable for skin avulsion imaging. On the other hand, the peptide GK is a caspase-3 recognizable peptide linker with the specific site DEVD and is considered as a specific peptide sequence that can be used to quantitatively detect the activity of caspase-3 in apoptotic cells (Yan et al., 2014).

**Scheme 1:**
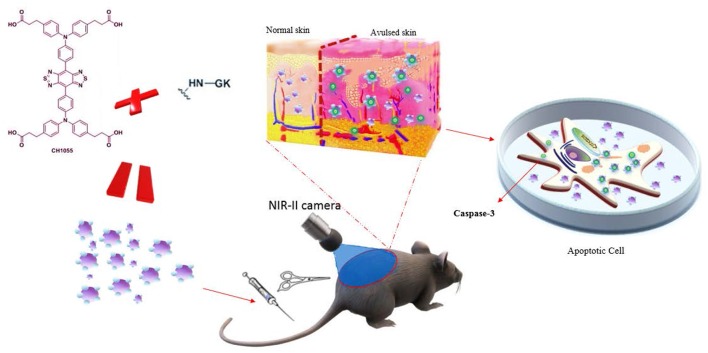
Schematic of GK-based NIR-II probe (CH1055-GK) binding to caspase-3 during the progression of skin avulsion in live mice and in apoptotic cells. The probe was diffused into avulsed skin after tail-vein injection and consistently retained in the skin. The probe then specifically bound to caspase-3 in apoptotic cells.

Based on these findings, we hypothesized that a small-molecule fluorescent dye CH1055 conjugated with peptide GK would target caspase-3 and visually reflect skin avulsion as well as necrotic regions (Scheme 1).

### Synthesis and Characterization of NIR-II Probes

**Chart 1:**
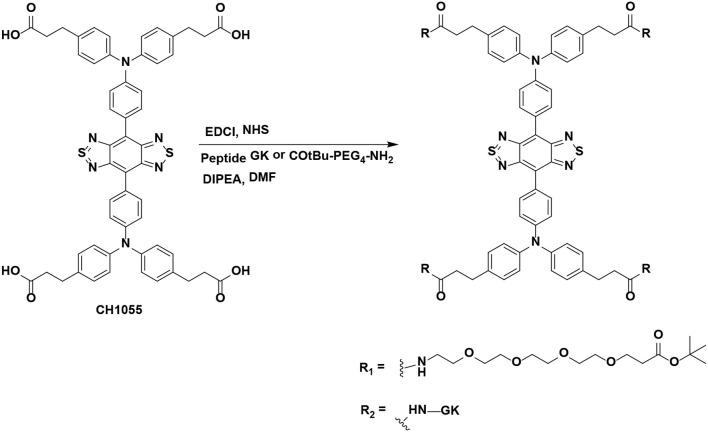
Chemical structure of CH1055-GK and control CH1055-PEG.

**Figure 1:**
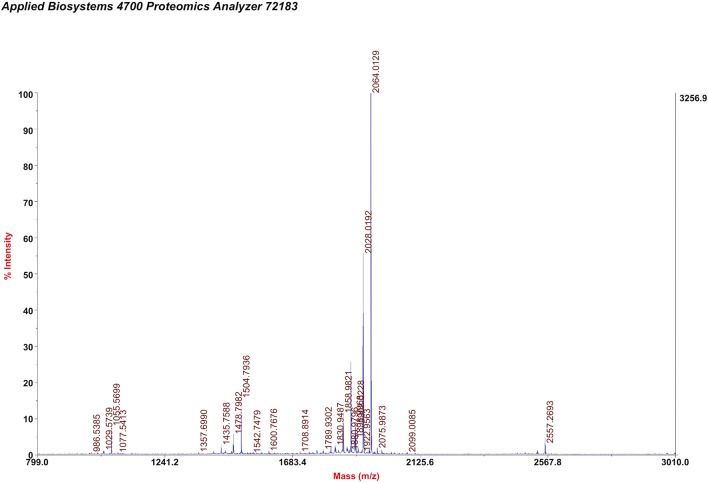
Measured MALDI-TOF-MS results for CH1055-GK.

NIR-II probes CH1055-GK and CH1055-PEG (as a control) were synthesized with CH1055 and the peptide GRRRDEVDK or its control COtBu-PEG4-NH2 (Chart 1 and Figure S1). As reported in our previous work, the NIR absorption bands of CH1055-WYRGRL (CH1055-WL) exhibited high stability and photostability, which would allow the probe to emit stable fluorescence in living organisms for a long time (Yi et al., 2019). Similarly, in this study, we designed the CH1055-GK probe, and the MALDI-TOF-MS measurement result for CH1055-GK was 2.064 KDa, while the expected M.W. is 2.063 KDa (Figure 1). Furthermore, the measured result of MALDI-TOF-MS for CH1055-PEG was 1.958 KDa, while the expected M.W. is 1.957 KDa (Figure S2).

### Probe Cell Uptakes and Binding Affinity in Apoptotic Cells

We performed cell uptakes and binding affinity tests of the small-molecule probe (CH1055-GK) with three apoptotic cells (HACAT, HUCEC, and HSF).

**Figure 2:**
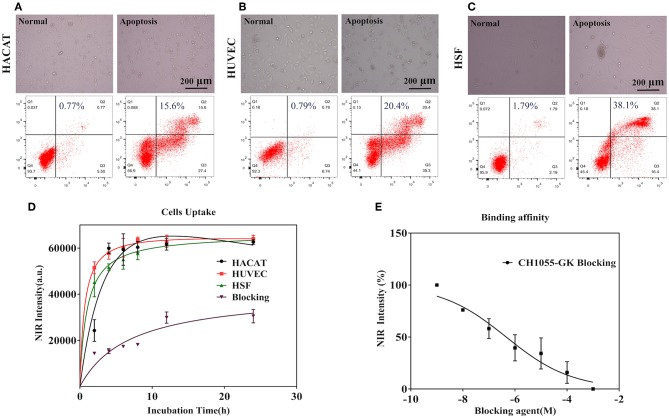
**(A–C)** Observation of cell morphology under a light microscope and representative flow cytometry results in normal (left) and apoptotic cells (right). **(D)** Cell uptakes and binding affinity of the NIR-II probe (CH1055-GK) in apoptotic cells (HACAT, HUVEC, and HSF) at different incubation times (0–24 h). **(E)** The binding affinity of CH1055-GK was measured using peptide GK (1 × 10^9^-1 × 10^3^ M) as the blocking agent. Mean ± SD, x-axis: incubation time (h) and blocking agent (M), y-axis: average NIR-II fluorescence intensity of the apoptotic cells.

As shown in the images displayed at the top in Figures 2A–C, normal and apoptotic cells had different cell morphologies under light microscope. In addition, the results of flow cytometry revealed that Adriamycin induced apoptosis (Q_2_ plus Q_3_) in 43% of HACAT, 55.7% of HUVEC, and 54.5% of HSF. Figure 2D shows that the NIR intensity of HUVEC and HSF significantly increased within 2 h and that a strong signal could be observed at 2 h (5.63 ± 0.259 × 10^4^) (4.52 ± 0.629 × 10^4^). After that, the fluorescence intensity continued to increase gradually until reaching a plateau at 12 h (6.29 ± 0.272 × 10^4^) (6.21 ± 0.095 × 10^4^). For the fluorescence intensity of HACAT, the fastest increase occurred during the period 2–4 h so that a strong signal could be detected at 4 h (5.99 ± 0.683 × 10^4^), and then fluorescence intensity similarly continued to increase until reaching the summit at 12 h (6.17 ± 0.098 × 10^4^). By contrast, while co-incubating with blocking agent (GK), the fluorescence intensity increased slowly, and the maximum intensity was 50% lower than that without blocking agent. The binding affinity assay was then performed by using different concentrations of the blocking peptide GK, after which the fluorescence intensity of cells significantly decreased when the concentrations of the blocking agent were increased before adding CH1055-GK (Figure 2E). In short, our results mainly demonstrated that probe CH1055-GK is so efficient for caspase-3 targeting because there are high accumulations of CH1055-GK in apoptotic cells, and such high uptakes were obviously reduced by the blocking agent.

### NIR-II Imaging of Skin Avulsion *in vivo* and *ex vivo*

**Figure 3:**
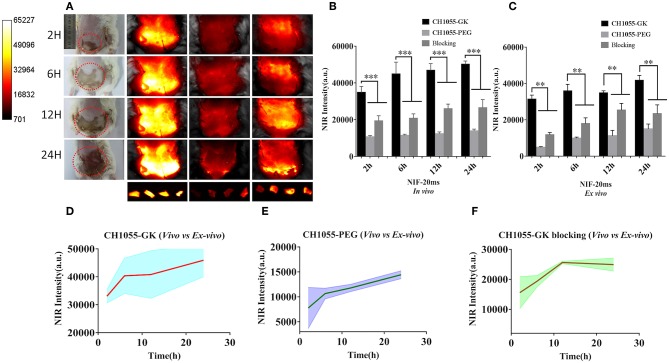
NIR-II imaging of CH1055-GK, PEGylated CH1055, and the blocking agent *in vivo* and *ex vivo*. **(A)** Representative photographs and NIR-II images (CH1055-GK, CH1055-PEG, and Blocking) at 2, 6, 12, and 24 h after tail-vein injection in skin avulsion models. *Ex vivo* imaging was also carried out at the same time intervals. **(B,C)** Quantitative comparison of NIR intensity in the avulsed skin of different groups. *N* = 6, mean ± SD. ***P < 0.001, **P < 0.01. The x-axis indicates time intervals, and the y-axis is fluorescent intensity. **(D–F)** Comparison between mean fluorescent intensity *in vivo* and *ex vivo*. The x-axis represents time intervals; the y-axis is the average of fluorescent intensity counts. 80 mW cm^−2^, 20 ms, LP 1000.

Based on photographs of skin avulsion in the mouse models, the irregular necrotic area (1 × 2 cm) in the avulsed skin of mice was observed at 2 h, and the necrotic area was expanded to 4 × 4 cm at 24 h (indicated by the red circle in Figure 3A). Interestingly, NIR images of CH1055-GK showed that fluorescence intensity gradually increased from 2 to 24 h post-injection and that the detected regions were wider in comparison with corresponding photographs, which meant that CH1055-GK not only allowed the rapid and accurate detection of injuries in the avulsed skin but assisted in distinctly observing the potential necrotic regions (Figures 3A,B, *p* < 0.001). In addition, it was noteworthy that control groups (CH1055-PEG) exhibited a much lower fluorescence intensity from 2 to 24 h and that blocking groups (CH1055-GK plus peptide GK) had a slower fluorescent signal growth trend than experimental groups (Figures 3A,B, *p* < 0.001). For *ex vivo* images of skin avulsion, statistical results were also obtained from 2 to 24 h and were almost consistent with our *in vivo* study, which indicated that CH1055-GK could penetrate into skin tissue and perform accurate detection in *ex-vivo* model of avulsed skin (Figures 3A,C, *p* < 0.01). As shown in Figures 3D–F, although differences could be observed between fluorescence intensity *in vivo* and *ex-vivo*, these results mutually supported the hypothesis that CH1055-GK can bind to caspase-3 for detecting skin avulsion and necrotic skin. Overall, our results suggested that the non-invasive small-molecule probe CH1055-GK provides a significant breakthrough in the detection of skin avulsion and necrosis in skin avulsion mouse models. Impressively, the potential necrotic regions in avulsed skin could also be detected precisely so that NIR-II imaging may provide assistance while determining necrotic regions in avulsed skins.

### Comparison of Skin-Avulsion Detection by NIR and MRI Imaging

**Figure 4:**
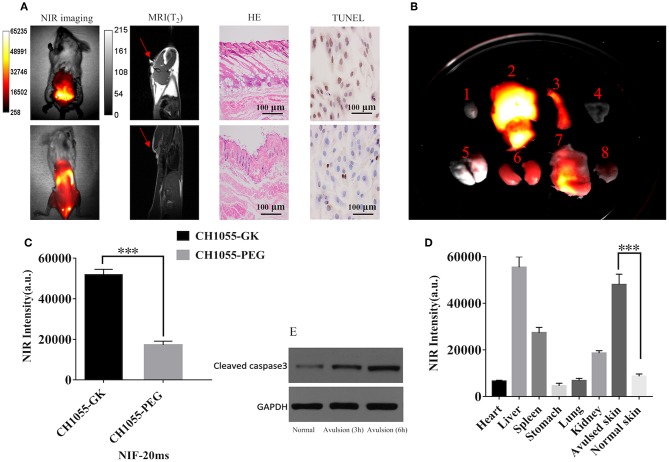
Evaluation of skin avulsion detection via NIR and MRI imaging. Images were taken in a supine position. **(A)** NIR images of CH1055-GK in skin avulsion and normal skin; T2-weighted MRI analysis of skin avulsion and normal skin; H&E or TUNEL staining of skin avulsion and normal skin. **(B)** Distribution of fluorescence intensity in the following organs (indicated by number): 1, heart; 2, liver; 3, spleen; 4, stomach; 5, lung; 6, kidney; 7, avulsed skin; 8, normal skin (*N* = 2). **(C)** Quantitative comparison of fluorescence intensity in skin avulsion and normal skin. *N* = 2, mean ± SD, ***p < 0.001. **(D)** Quantitative comparison of fluorescence intensity in the following organs (indicated by number in **B**): 1, heart; 2, liver; 3, spleen; 4, stomach; 5, lung; 6, kidney; 7, avulsed skin; 8, normal skin (*N* = 2). ***p < 0.001. **(E)** Western blot analysis indicated the expression of caspase-3 in normal and avulsed skin. 80 mW cm^−2^, 20 ms, LP 1000. Scale bar, 100 μ*m*.

The results of *in vivo* NIR imaging demonstrated increased fluorescence intensity of CH1055-GK in necrotic regions of skin avulsion compared with normal skin (Figures 4A,C, *p* < 0.001). Like NIR-II imaging, MRI analysis could identify the ruptured skin and verify some palpable differences between the avulsed and the normal skin, but it was difficult to determine the necrotic regions in the early stage of skin avulsion (Figure 4A, red arrow). Additionally, H&E staining indicated that there were obvious biological differences in avulsed skin compared with normal skin, such as disarrangement of skin tissue structure (Figure 4A). Furthermore, TUNEL positive stained cells showed an increase in apoptotic cells in avulsed skin (Figure 4A). Western blotting analysis also showed high expression of active caspase-3 in avulsed skin at 3 h and 6 h (Figure 4E). After the probe was administered into the mice via tail-vein injection, high retention of CH1055-GK appeared in avulsed skin as well as in other organs including the liver and spleen (Figures 4C,D, *p* < 0.001). Taken together, these results not only indicate that apoptosis might lead to irreversible damage in skin avulsion and result in necrosis but also showed that the caspase-3 targeting NIR-II probe (CH1055-GK) could better visually detect early skin avulsion and necrosis in skin avulsion mouse models compared to typical MRI analysis. In our study, high or overexpression of caspase-3 could recognize GK peptide, and caspase-7 might also be able to recognize this peptide, which will be clarified in further studies.

### Immunofluorescence Staining for Specific Binding of CH1055-GK to Caspase-3 in Apoptotic Cells

**Figure 5:**
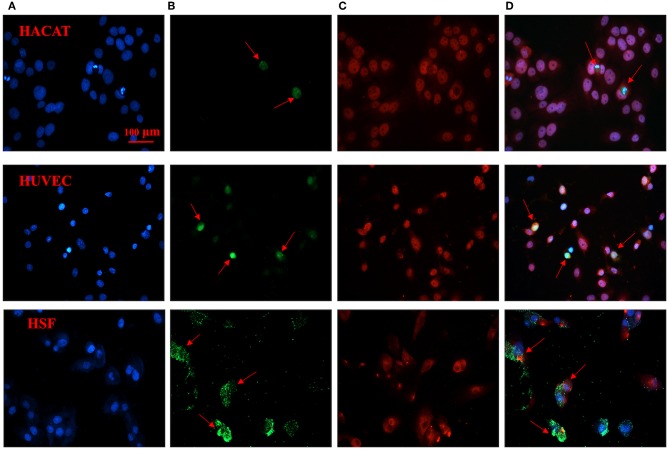
Immunofluorescence staining of CH1055-GK binding to caspase-3 in apoptotic cells. The following immunofluorescent signals are shown: **(A)** blue fluorescence staining represents the nucleus; **(B)** green fluorescence signals show the FITC-labeled probe (FITC-CH1055-GK); **(C)** red fluorescence signals reveal the combination of caspase-3 with mouse anti-human antibody and of Cy3 with goat anti-mouse secondary antibody; **(D)** superimposed images. Scale bar, 100 μ*m*.

Our immunofluorescence staining results validated the binding affinity of probe CH1055-GK to caspase-3. The green fluorophore dye (FITC) was used to label CH1055-GK, and the red fluorophore dye (Cy3) with goat anti-mouse secondary antibody was applied to label caspase-3 (Figures 5A–C). While co-incubating with FITC-CH1055-GK and caspase-3-Cy3, apoptotic cells with high expression or overexpression of caspase-3 could also display green fluorescence, which indicated that the probe was bound to caspase-3 (Figure 5D, red arrows). In contrast, non-apoptotic cells could not show green fluorescence. Therefore, our immunofluorescence staining results indicated that the probe could be specifically bound to caspase-3 in apoptotic cells.

### *In vitro* Cytotoxicity and *in vivo* Toxicity

**Figure 6:**
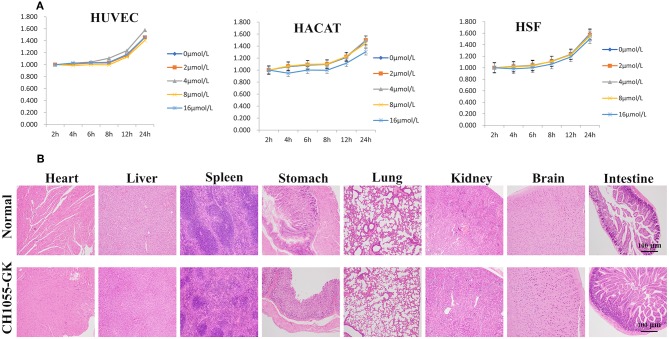
Cytotoxicity of CH1055-GK *in vitro* and *in vivo*. **(A)** Cytotoxicity of CH1055-GK was evaluated by CCK-8 assay in human cells (HUVEC, HSF, and HACAT). **(B)** H&E staining of organs (Heart, Liver, Spleen, Stomach, Lung, Kidney, Brain, and Intestine) in normal and CH1055-GK groups. Scale bar, 100 μ*m*.

The *in vitro* cytotoxicity of CH1055-GK was assayed in the human cell lines (HACAT, HUVEC, and HSF). According to the results, there was no obvious cytotoxicity in these normal cells incubated with different concentrations of the probe for 2–24 h (Figure 6A). For *in vivo* toxicity, CH1055-GK was administered to the mice via tail-vein injection at a dosage of 10 mg kg^−1^. The results of H&E staining of the main organs (Heart, Liver, Spleen, Stomach, Lung, Kidney, Brain, and Intestine) indicated that there was no diagnostic damage or inflammation in normal skin compared with the control group (Figure 6B). Although biocompatibility and biosafety were not investigated in our study, CH1055-GK displayed a potential for early detection of skin avulsion and determination of skin necrosis.

## Conclusion

In summary, we have synthesized a novel NIR-II fluorescent probe CH1055-GK, and our results proved that the probe emitted high levels of fluorescence in apoptotic cells and in skin avulsion models *in vivo* and *ex vivo*, which indicated that CH1055-GK had strong binding affinity for caspase-3. We hypothesize that this probe is a promising early skin avulsion diagnostic tool and can be used to differentiate potential necrotic regions from surrounding healthy or inflammatory tissues.

Our future studies will be devoted to consistently exploring the possible application of this probe to the diagnosis of skin avulsion in the early stages and in therapeutic monitoring in the clinic. Certainly, the toxicity of the probe *in vivo* and the study of diffusion kinetics should be further investigated before the probe can be admitted for use in clinical trials.

## Data Availability Statement

All datasets generated for this study are included in the article/Supplementary Material.

## Ethics Statement

All animal experiments were approved by the Wuhan University Animal Care and Use Committee and carried out according to relevant national and international guidelines. The panel's specific instructions regarding care, treatment, and euthanasia were strictly followed for the animals used in this study. The IACUC number is 2019149.

## Author Contributions

YL was responsible for performing the experiments and writing the manuscript. WY, DL, and YG were responsible for providing cells. XH and BQ were responsible for discussing the experimental results. AY was responsible for designing experiments and revising the paper.

### Conflict of Interest

The authors declare that the research was conducted in the absence of any commercial or financial relationships that could be construed as a potential conflict of interest.

## References

[B1] Antaris A. L. Chen H. Cheng K. Sun Y. Hong G. Qu C.. (2016). A small-molecule dye for NIR-II imaging. Nat. Mater. 15, 235–242. 10.1038/nmat4476 26595119

[B2] Antaris A. L. Chen H. Diao S. Ma Z. Zhang Z. Zhu S.. (2017). A high quantum yield molecule-protein complex fluorophore for near-infrared II imaging. Nat. Commun. 8:15269. 10.1038/ncomms15269 28524850 PMC5454457

[B3] Ballestin A. Casado J. G. Abellan E. Vela F. J. Alvarez V. Uson A.. (2018). Ischemia-reperfusion injury in a rat microvascular skin free flap model: a histological, genetic, and blood flow study. PLoS ONE 13:e0209624. 10.1371/journal.pone.0209624 30589864 PMC6307726

[B4] Casares N. Pequignot M. O. Tesniere A. Ghiringhelli F. Roux S. Chaput N.. (2005). Caspase-dependent immunogenicity of doxorubicin-induced tumor cell death. J. Exp. Med. 202, 1691–1701. 10.1084/jem.20050915 16365148 PMC2212968

[B5] Chin T. Y. Kiat S. S. Faizul H. G. Wu W. Abdullah J. M. (2017). The effects of minocycline on spinal root avulsion injury in rat model. Malays. J. Med. Sci. 24, 31–39. 10.21315/mjms2017.24.1.4 28381927 PMC5346001

[B6] Crowley L. C. Marfell B. J. Scott A. P. Waterhouse N. J. (2016). Triggering apoptosis in hematopoietic cells with cytotoxic drugs. Cold Spring Harb. Protoc. 2016, 635–638. 10.1101/pdb.prot087130 27371592

[B7] Gagnier J. J. Kienle G. Altman D. G. Moher D. Sox H. Riley D. (2013). The CARE guidelines: consensus-based clinical case reporting guideline development. Glob. Adv. Health Med. 2, 38–43. 10.7453/gahmj.2013.008 24416692 PMC3833570

[B8] Hong G. Diao S. Antaris A. L. Dai H. (2015). Carbon nanomaterials for biological imaging and nanomedicinal therapy. Chem. Rev. 115, 10816–10906. 10.1021/acs.chemrev.5b00008 25997028

[B9] Hong G. Robinson J. T. Zhang Y. Diao S. Antaris A. L. Wang Q.. (2012). *In vivo* fluorescence imaging with Ag2S quantum dots in the second near-infrared region. Angew. Chem. Int. Ed Engl. 51, 9818–9821. 10.1002/anie.201206059 22951900

[B10] Hong G. Zou Y. Antaris A. L. Diao S. Wu D. Cheng K.. (2014). Ultrafast fluorescence imaging *in vivo* with conjugated polymer fluorophores in the second near-infrared window. Nat. Commun. 5:4206. 10.1038/ncomms5206 24947309

[B11] Iglesias M. Butron P. Leon-Lopez D. A. Garcia-Mancilla S. Espino-Gaucin I. Rubio A. (2014). Soft tissue reconstruction with omental free flap in complex upper extremity injuries: report of 13 cases. Microsurgery 34, 425–433. 10.1002/micr.22236 24523014

[B12] Latifi R. El-Hennawy H. El-Menyar A. Peralta R. Asim M. Consunji R.. (2014). The therapeutic challenges of degloving soft-tissue injuries. J. Emerg. Trauma Shock 7, 228–232. 10.4103/0974-2700.136870 25114435 PMC4126125

[B13] Li T. Li C. Ruan Z. Xu P. Yang X. Yuan P.. (2019). Polypeptide-conjugated second near-infrared organic fluorophore for image-guided photothermal therapy. ACS Nano 13, 3691–3702. 10.1021/acsnano.9b00452 30790523

[B14] Lin J. Zeng X. Xiao Y. Tang L. Nong J. Liu Y.. (2019). Novel near-infrared II aggregation-induced emission dots for *in vivo* bioimaging. Chem. Sci. 10, 1219–1226. 10.1039/c8sc04363a 30774922 PMC6349025

[B15] Liu Y. Q. Liu Y. F. Ma X. M. Xiao Y. D. Wang Y. B. Zhang M. Z.. (2015). Hydrogen-rich saline attenuates skin ischemia/reperfusion induced apoptosis via regulating Bax/Bcl-2 ratio and ASK-1/JNK pathway. J. Plast. Reconstr. Aesthet. Surg. 68, e147–e156. 10.1016/j.bjps.2015.03.001 26003800

[B16] MacFarlane D. Shah K. Wysong A. Wortsman X. Humphreys T. R. (2017). The role of imaging in the management of patients with nonmelanoma skin cancer: diagnostic modalities and applications. J. Am. Acad. Dermatol. 76, 579–588. 10.1016/j.jaad.2015.10.010 28325388

[B17] Milcheski D. A. Nakamoto H. A. Tuma P. Jr. Nobrega L. Ferreira M. C. (2013). Experimental model of degloving injury in rats: effect of allopurinol and pentoxifylline in improving viability of avulsed flaps. Ann. Plast. Surg. 70, 366–369. 10.1097/SAP.0b013e318230601a 21921788

[B18] Ntziachristos V. Ripoll J. Wang L. V. Weissleder R. (2005). Looking and listening to light: the evolution of whole-body photonic imaging. Nat. Biotechnol. 23, 313–320. 10.1038/nbt1074 15765087

[B19] Olingy C. E. San Emeterio C. L. Ogle M. E. Krieger J. R. Bruce A. C. Pfau D. D.. (2017). Non-classical monocytes are biased progenitors of wound healing macrophages during soft tissue injury. Sci. Rep. 7:447. 10.1038/s41598-017-00477-1 28348370 PMC5428475

[B20] Pansare V. Hejazi S. Faenza W. Prud'homme R. K. (2012). Review of long-wavelength optical and NIR imaging materials: contrast agents, fluorophores and multifunctional nano carriers. Chem. Mater. 24, 812–827. 10.1021/cm2028367 22919122 PMC3423226

[B21] Ponder K. G. Boise L. H. (2019). The prodomain of caspase-3 regulates its own removal and caspase activation. Cell Death Discov. 5:56. 10.1038/s41420-019-0142-1 30701088 PMC6349851

[B22] Reinsmith L. E. Garcia-Elias M. Gilula L. A. (2013). Traumatic axial dislocation injuries of the wrist. Radiology 267, 680–689. 10.1148/radiol.13111682 23704291

[B23] Rogers C. Fernandes-Alnemri T. Mayes L. Alnemri D. Cingolani G. Alnemri E. S. (2017). Cleavage of DFNA5 by caspase-3 during apoptosis mediates progression to secondary necrotic/pyroptotic cell death. Nat. Commun. 8:14128. 10.1038/ncomms14128 28045099 PMC5216131

[B24] Sakai G. Suzuki T. Hishikawa T. Shirai Y. Kurozumi T. Shindo M. (2017). Primary reattachment of avulsed skin flaps with negative pressure wound therapy in degloving injuries of the lower extremity. Injury 48, 137–141. 10.1016/j.injury.2016.10.026 27788928

[B25] Scabini M. Stellari F. Cappella P. Rizzitano S. Texido G. Pesenti E. (2011). *In vivo* imaging of early stage apoptosis by measuring real-time caspase-3/7 activation. Apoptosis 16, 198–207. 10.1007/s10495-010-0553-1 21082356

[B26] Sen H. Oruc M. Isik V. M. Sadic M. Sayar H. Citil R.. (2017). The effect of omeprazole usage on the viability of random pattern skin flaps in rats. Ann. Plast. Surg. 78, e5–e9. 10.1097/sap.0000000000000922 27779491

[B27] Shaulov-Rotem Y. Merquiol E. Sadan T. W. Moshel O. Blum G. J. C. S. (2016). A novel quenched fluorescent activity-based probe reveals caspase-3 activity in the endoplasmic reticulum during apoptosis. Chem. Sci. 7, 1322–1337. 10.1039/C5SC03207E 29910890 PMC5975724

[B28] Siemionow M. Arslan E. (2004). Ischemia/reperfusion injury: a review in relation to free tissue transfers. Microsurgery 24, 468–475. 10.1002/micr.20060 15378577

[B29] Smith A. M. Mancini M. C. Nie S. (2009). Bioimaging: second window for *in vivo* imaging. Nat. Nanotechnol. 4, 710–711. 10.1038/nnano.2009.326 19898521 PMC2862008

[B30] Srinivasula S. M. Hegde R. Saleh A. Datta P. Shiozaki E. Chai J.. (2001). A conserved XIAP-interaction motif in caspase-9 and Smac/DIABLO regulates caspase activity and apoptosis. Nature 410, 112–116. 10.1038/35065125 11242052

[B31] Strzyz P. (2017). Cell death: pulling the apoptotic trigger for necrosis. Nat. Rev. Mol. Cell Biol. 18:72. 10.1038/nrm.2017.1 28074061

[B32] Sun S. Inoue K. Y. Shiomoto S. Takano S. Matsue T. J. C. (2017a). Amperometric detection of apoptosis by using p-methoxyaniline-conjugated substrate for caspase-3. ChemElectroChem 4, 941–946. 10.1002/celc.201600700

[B33] Sun Y. Ding M. Zeng X. Xiao Y. Wu H. Zhou H.. (2017b). Novel bright-emission small-molecule NIR-II fluorophores for *in vivo* tumor imaging and image-guided surgery. Chem. Sci. 8, 3489–3493. 10.1039/c7sc00251c 28507722 PMC5418643

[B34] Sun Y. Qu C. Chen H. He M. Tang C. Shou K.. (2016). Novel benzo-bis(1,2,5-thiadiazole) fluorophores for *in vivo* NIR-II imaging of cancer. Chem. Sci. 7, 6203–6207. 10.1039/c6sc01561a 30034761 PMC6024204

[B35] Sun Y. Zeng X. Xiao Y. Liu C. Zhu H. Zhou H.. (2018). Novel dual-function near-infrared II fluorescence and PET probe for tumor delineation and image-guided surgery. Chem. Sci. 9, 2092–2097. 10.1039/c7sc04774f 29675250 PMC5892408

[B36] Taylor R. C. Cullen S. P. Martin S. J. (2008). Apoptosis: controlled demolition at the cellular level. Nat. Rev. Mol. Cell Biol. 9, 231–241. 10.1038/nrm2312 18073771

[B37] Tosti R. Eberlin K. R. (2018). “Damage Control” hand surgery: evaluation and emergency management of the Mangled Hand. Hand Clin. 34, 17–26. 10.1016/j.hcl.2017.09.002 29169593

[B38] Ueno N. T. Espinosa Fernandez J. R. Cristofanilli M. Overmoyer B. Rea D. Berdichevski F.. (2018). International consensus on the clinical management of inflammatory breast cancer from the Morgan Welch Inflammatory Breast Cancer Research Program 10th Anniversary Conference. J. Cancer 9, 1437–1447. 10.7150/jca.23969 29721054 PMC5929089

[B39] Walton E. Williams M. Robinson J. R. (2017). Incarceration of the medial collateral ligament in the intercondylar notch following proximal avulsion. Skeletal Radiol. 46, 1575–1578. 10.1007/s00256-017-2723-5 28730295

[B40] Weinand C. (2018). Degloving injuries of upper extremity: a strategy with full thickness skin mesh. World J. Plast. Surg. 7, 372–376. 10.29252/wjps.7.3.372 30560080 PMC6290308

[B41] Wu L. Hu Y. He Y. Xia Y. Lu H. Cao Z.. (2019). Dual-channel surface plasmon resonance monitoring of intracellular levels of the p53-MDM2 complex and caspase-3 induced by MDM2 antagonist Nutlin-3. Analyst 144, 3959–3966. 10.1039/c9an00301k 31134974

[B42] Yan H. He L. Zhao W. Li J. Xiao Y. Yang R.. (2014). Poly beta-cyclodextrin/TPdye nanomicelle-based two-photon nanoprobe for caspase-3 activation imaging in live cells and tissues. Anal. Chem. 86, 11440–11450. 10.1021/ac503546r 25347212

[B43] Yang J. Xie Q. Zhou H. Chang L. Wei W. Wang Y.. (2018). Proteomic analysis and NIR-II imaging of MCM2 protein in hepatocellular carcinoma. J. Proteome Res. 17, 2428–2439. 10.1021/acs.jproteome.8b00181 29750532

[B44] Yang Q. Ma Z. Wang H. Zhou B. Zhu S. Zhong Y.. (2017). Rational design of molecular fluorophores for biological imaging in the NIR-II window. Adv. Mater. 29:1605497. 10.1002/adma.201605497 28117499

[B45] Yi W. Zhou H. Li A. Yuan Y. Guo Y. Li P.. (2019). A NIR-II fluorescent probe for articular cartilage degeneration imaging and osteoarthritis detection. Biomater. Sci. 7, 1043–1051. 10.1039/c8bm01440j 30628591

[B46] Zeng X. Chen Z. Tang L. Yang H. Liu N. Zhou H.. (2019). A novel near-infrared fluorescent light-up probe for tumor imaging and drug-induced liver injury detection. Chem. Commun. (Camb). 55, 2541–2544. 10.1039/c8cc10286d 30742156

[B47] Zeng X. Xiao Y. Lin J. Li S. Zhou H. Nong J.. (2018). Near-infrared II dye-protein complex for biomedical imaging and imaging-guided photothermal therapy. Adv. Healthc. Mater. 7:e1800589. 10.1002/adhm.201800589 30051654

[B48] Zhang X. D. Wang H. Antaris A. L. Li L. Diao S. Ma R.. (2016). Traumatic brain injury imaging in the second near-infrared window with a molecular fluorophore. Adv. Mater. 28, 6872–6879. 10.1002/adma.201600706 27253071 PMC5293734

