# Supplementary material for: NIR-II Fluorescence Imaging of Skin Avulsion and Necrosis
Source: Front Chem. 2019 Oct 22;7:696. doi: 10.3389/fchem.2019.00696 (PMC6817597; doi:10.3389/fchem.2019.00696)
Supplement: Supplementary file 1 [file Data_Sheet_1.docx]

NIR-II Fluorescence Imaging of Skin Avulsion and Necrosis

Yizhou Li,^a^ Xiang Hu,^a^ Wanrong Yi,^a^ Daifeng Li,^a^ Yaqi Guo,^a^ Baiwen Qi,^a^ Aixi Yu*^a^

^a^ Department of Orthopedics Trauma and Microsurgery, Zhongnan Hospital of Wuhan University, Hubei Province, Wuhan 430071, China.

Postal address: Department of Orthopedics Trauma and Microsurgery, Zhongnan Hospital of Wuhan University, Number 169, East Lake Road, Wuhan, Hubei 430071, China

* Corresponding author. Department of Orthopedics Trauma and Microsurgery, Zhongnan Hospital of Wuhan University, Hubei Province, Wuhan 430071, China.

Email: [yuaixi@whu.edu.cn](mailto:yuaixi@whu.edu.cn)

**Experiment**

**1 Synthesis of CH1055-PEG or CH1055-GK**

a) Synthesis of CH1055 can be referred to the previous literature(Antaris et al., 2016). Synthesis of CH1055-GK or CH1055-PEG

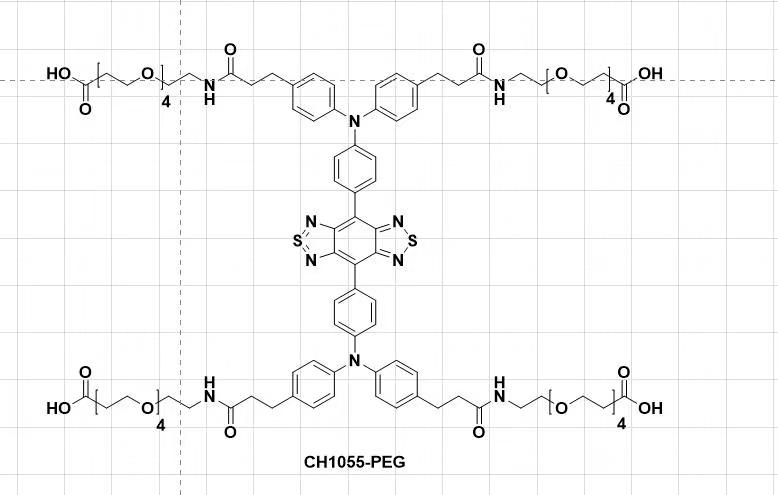


Figure S1. Synthesis of **CH1055-GK** and **CH1055-PEG**.

All chemicals reagents were purchased from commercial sources (such as Aldrich, Ameresco, Energy Chemical) and used without further purification unless otherwise noted. HPLC was performed on a Dionex HPLC System (Dionex Corporation) equipped with a GP50 gradient pump and an in-line diode array UV-Vis detector. The peptide GK (1112.21dpa) were purchased from GL Biochem (Shanghai) Ltd. Synthesis of CH1055 (969dpa) can be referred to the previous literature(Antaris et al., 2016). Synthesis of CH1055-GK or CH1055-PEG (as a control): to a solution of CH1055 (100 μg, 0.103 μmol) in dry DMF (N,N-Dimethylformamide) was stirred for 10 min, NHS (N-hydroxysuccinimide) (59.27 μg, 0.515 μmol) and EDCI (1-ethyl-3-(3- dimethylaminopropyl) carbodiimide) (98.73 μg, 0.515 μmol) were added in the mixture and stirred for 2 h under N_2_ protection. Then, peptide GK (581.44 μg, 0.515 μmol) or COtBu-PEG_4_-NH2 (Polyethylene glycol, 1000dpa, Ponsure Biological) (165.52 μg, 0.515 μmol) and DIPEA (N, N-diisopropylethylamine) (15 μL) was added in the reaction solutions and stirred overnight. The final product was purified by HPLC. HPLC condition: eluent: water/acetonitrile (containing 0.1% TFA, from 70% to 95%); over 22 min with a flow rate of 1 mL min−1 and detection at 254 nm.” The final product CH1055-GK or CH1055-PEG was confirmed using MALDI-TOF-MS.

b) The final product CH1055-PEG was confirmed using MALDI-TOF-MS.
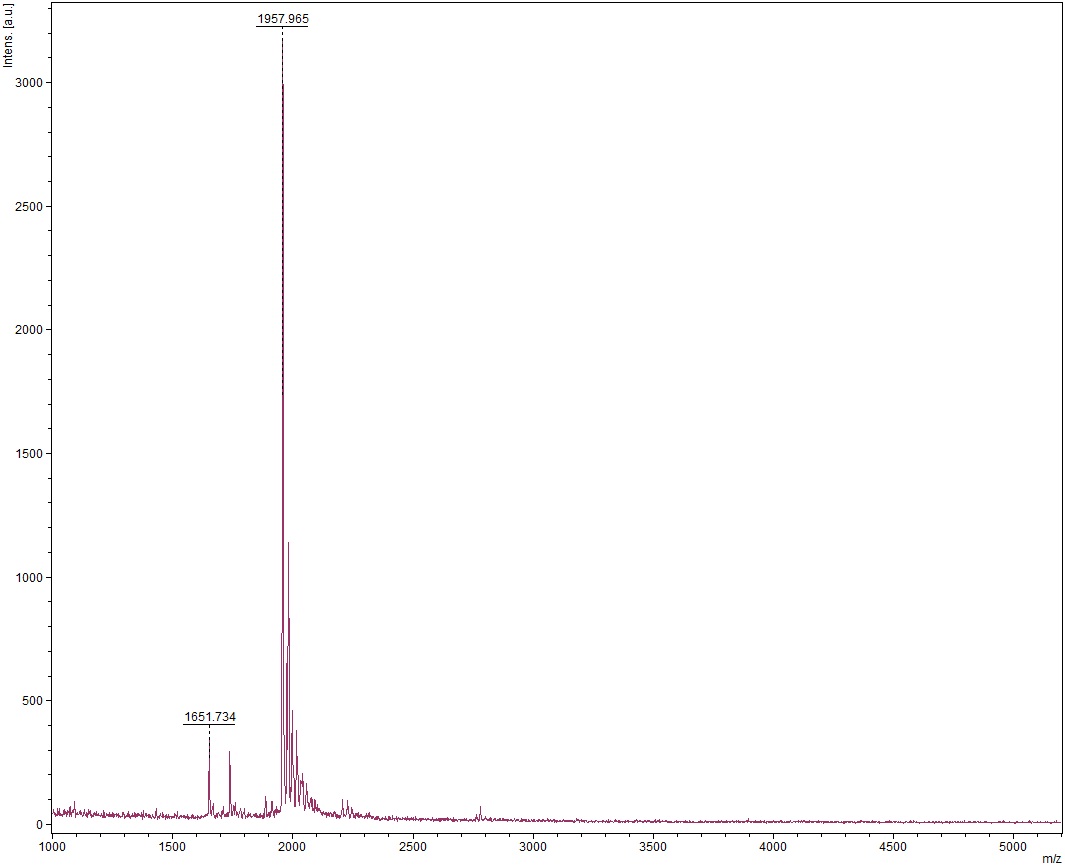


Figure S2. Results of measured MALDI-TOF-MS for CH1055-PEG. The result of measured MALDI-TOF-MS for CH1055-PEG was 1.958KDa and the expected M.W. is 1.957KDa.

**2 Apoptosis**

**1) Apoptosis induction**

For the apoptosis induction, cells (HUVEC, HSF, HACAT) were cultured in 6-wells for 24 hours and then 2 mL of culture medium mixed with 20 μmol/L Adriamycin (Thermo Fisher Scientific Inc) was added to each 6-wells and cultured for 6 and 12h at 37 °C incubator with 5% CO_2_(Crowley et al., 2016).

**2) Flow cytometric analysis**

The collected cells were prepared for the flow cytometer as follows:

a) Pre-cool PBS to 4 °C and dilute appropriate amount of Binding Buffer for preparation.

b) Collecting the culture solution in a flow tube. The cells in the six-well plate are washed twice with PBS, and 0.5 mL of 0.25% trypsin-digested cells are added. After the cells are rounded and some cells are detached, the medium is added to terminate the digestion.

c) We gently pipette the cells into a flow tube. The tubes are centrifuged at 300rpm for 5min and the supernatant was discarded.

d) Resuspend the cells by adding 1 mL of PBS. Cells were centrifugation at 300 rpm for 5 min and the supernatant was discarded.

e) Cells were harvested and resuspended in 300 μl binding buffer containing 5 μl Annexin V-FITC (Fluorescein Isothiocyanate, Sungene Biotech) as well as 5 μl PI (Propidium Iodide, Sungene Biotech), and then were incubated at room temperature (RT) in the dark for 15min

f) we ran each cell sample and the data was quantified by flow cytometer.

**3 Immunofluorescence**

**1) Groups**

A Control

B Control + Adriamycin (20 μM)

C Control + Adriamycin (20 μM) + Probe (150μM)

**2) Detection indicators and corresponding antibody information:**

| **Product** | **Primary antibody** | **manufacturer** | **Number** | **Dilution** |
| --- | --- | --- | --- | --- |
| Cleaved Caspase-3 | Rabbit | ABCAM | ab49822 | 1:1000 |
| **Secondary antibody** | |  | **Number** | **Dilution** |
| CY3 Goat anti-rabbit | | ASPEN | AS-1109 | 1:50 |

**4 Western blotting**

**1). Extraction of proteins from tissues**

a) Dissect the tissue of interest on ice, and as quickly as possible to prevent degradation by proteases.

b) Place the tissue in round-bottom microcentrifuge tubes and immerse in liquid nitrogen to snap freeze. Store samples at -80°C for later use. For a ~3 mg piece of tissue, add ~300 μL of ice cold lysis buffer rapidly to the tube, homogenize with an electric homogenizer, rinse the blade twice with another 2 x 200 μL lysis buffer, then maintain constant agitation for 2 h at 4°C. Volumes of lysis buffer must be determined in relation to the amount of tissue present.

c) Centrifuge for 20 min at 13,000 rpm at 4°C in a microcentrifuge. Gently remove the tubes from the centrifuge and place on ice, aspirate the supernatant and place in a fresh tube kept on ice; discard the pellet.

**2). Normalize total protein concentration**

Remove a small volume of lysate to perform a protein quantification assay. Determine the protein concentration for each cell lysate using a BCA protein concentration assay kit.

**3). SDS-PAGE gel electrophoresis**

a) Load equal amounts of protein into the wells of the SDS-PAGE gel, along with molecular weight marker. Load 20–30 μg of total protein from tissue homogenate, or 10–100 ng of purified protein.

b) Constant pressure electrophoresis was carried out according to concentrated gel 80V and separation gel 120V until the bromophenol blue reached the lower edge of the rubber sheet.

**4). Transferring the protein from the gel to the membrane**

a) Preparation of a transfer filter paper and PVDF membrane and the PVDF membrane must be activated with methanol before using.

b) Assemble the transfer sandwich and make sure no air bubbles are trapped in it. The blot should be on the cathode and the gel on the anode.

c) Transfer in at a constant current of 300MA and the transfer time can be adjusted by the molecular weight of the target protein.

**5). Antibody incubation**

a) Block the membrane for 1 h at room temperature using blocking buffer.

b) Incubate the membrane with appropriate dilutions of primary antibody in blocking buffer overnight incubation at 4°C

c) Recover the diluted primary antibody solution and wash three time with TBST for 5 min each time.

d) Incubate the membrane with the recommended dilution of conjugated secondary antibody in blocking buffer at room temperature for 1 h, and then wash the membrane in three washes of TBST, 5 min each.

**6). Imaging**

Add the freshly prepared ECL mixed solution to the protein side of the membrane and expose it in the dark room. The exposure conditions are adjusted according different light intensity.

**7). Data analysis**

The AlphaEaseFC software processing system analyzes the optical density of the target band.

**5 TUNEL staining**

a) the cell suspension was added to a glass coverslip and incubated at 37°C incubator with 5% CO_2_ for 2 h, followed by washing in PBS for 3 × 5 min.

b) Then cells were 4% paraformaldehyde for 30 min at RT and washed 3 times with PBS.

c) Next, cells were incubated with 50-100 μl permeabilization buffer (0.5%Triton X-100 in PBS) for 10 min at RT and washed 3 times with PBS for 5 min each. Cells were then incubated with 3% hydrogen peroxide solution for 20 min in the dark at RT, followed by three PBS washes.

d) the staining solution was prepared by mixing 1:9 dilution of the reagents (TdT: dUTP,) provided in the assay kit and incubate in the 37°C water bath for 60 min.

e) Add appropriate amount of reagent 3 (converter-POD) to each well to cover the cells. The slides were placed in a wet box and incubated at 37 ° C for 30 min.

f) The 3–3'-diaminobenzidine tetrahydrochloride (DAB) solution was added to each well and monitored under the microscope and lasted from 10 sec to 2 min. The color of the nucleus was brown.

g) After counterstaining with Harris’ hematoxylin, slides were dehydrated mounted on a glass slide with a neutral gum.

**Reference**

Antaris, A.L., Chen, H., Cheng, K., Sun, Y., Hong, G., Qu, C., et al. (2016). A small-molecule dye for NIR-II imaging. *Nat Mater* 15(2)**,** 235-242. doi: 10.1038/nmat4476.

Crowley, L.C., Marfell, B.J., Scott, A.P., and Waterhouse, N.J. (2016). Triggering Apoptosis in Hematopoietic Cells with Cytotoxic Drugs. *Cold Spring Harb Protoc* 2016(7). doi: 10.1101/pdb.prot087130.
